# A buffalo rumen-derived probiotic (SN-6) could effectively increase simmental growth performance by regulating fecal microbiota and metabolism

**DOI:** 10.3389/fmicb.2022.935884

**Published:** 2022-10-28

**Authors:** Shumin Yang, Ji Luo, Yingying Chen, Rui Wu, Huazhen Liu, Zutao Zhou, Muhammad Akhtar, Yuncai Xiao, Deshi Shi

**Affiliations:** ^1^State Key Laboratory of Agriculture Microbiology, College of Veterinary Medicine, Huazhong Agricultural University, Wuhan, China; ^2^Suining Mubiao Agricultural Development Co., Ltd., Xuzhou, China; ^3^Department of Basic Veterinary Medicine, College of Animal Science and Veterinary Medicine, Huazhong Agricultural University, Wuhan, China; ^4^Key Laboratory of Agricultural Animal Genetics, Breeding and Reproduction of Ministry of Education, Huazhong Agricultural University, Wuhan, China

**Keywords:** *Bacillus*, fiber decomposition, production performance, fecal microbiota, metabolomic

## Abstract

Microorganisms play a key role in ruminal digestion, some of which can be used as probiotics to promote growth in ruminants. However, which potential bacteria are responsible for ruminant growth and how they potentiate the basic mechanism is unclear. In this study, three bacterial strains, *Bacillus pumilus* (SN-3), *Bacillus paralicheniformis* (SN-6), and *Bacillus altitudinis* (SN-20) with multiple digestive enzymes were isolated from the rumen of healthy buffaloes. Among these strains, SN-6 secreted cellulase, laccase, and amylase, and significantly inhibited *Staphylococcus aureus* ATCC25923 and *Escherichia coli* K99 *in vitro.* In addition, SN-6 exhibited strong tolerance to artificial gastric juice, intestinal juice, and high temperature. Antibiotic resistance test, virulence gene test, and mouse toxicity test confirmed the safety of SN-6. Further, SN-6 significantly increased the body weight (*p* < 0.01), affects the intestinal microbiota structure, and alters the metabolomic patterns of Simmental. There was a remarkable difference in the β diversity of fecal microflora between SN-6 and control groups (*p* < 0.05). Furthermore, SN-6 significantly increased the abundance of *Clostridium_sensu_stricto_1*, *Bifidobacterium*, *Blautia*, and *Cellulolyticum*, decreased the relative abundance of *Monoglobus* and *norank_f_Ruminococcacea*. Moreover, SN-6 feeding significantly enriched intestinal metabolites (i.e., 3-indoleacrylic acid, kynurenic acid) to maintain intestinal homeostasis. Finally, the microbial and metabolic functional analysis indicated that SN-6 could enhance amino acid metabolism (mainly tryptophan metabolism) and lipid metabolism pathways. Overall, these findings indicated that SN-6 could be used as a probiotic in ruminants.

## Introduction

Ruminants play a significant role in our society because they were domesticated more than 10,000 years ago. They uniquely use a variety of digestive enzymes and digest the most complex polysaccharides, which are undigestible by the human digestive tract (Mizrahi et al., 2021). Such enzymes are predominantly produced by microbes of ruminants and are responsible for breaking down plant fibers (Russell and Rychlik, 2001; Jose et al., 2017). It is interesting to note that the microbiota in the gut of ruminants is diverse and abundant. That is why, the rumen, an important foregut fermenter with a strong capacity to digest plant feed, is strictly dependent on a complex array of gut microbes for its physiological and biochemical responses. These complex rumen microorganisms degrade plant fibers in ruminant roughage by expressing and secreting various digestive enzymes, i.e., cellulase, protease, amylase, etc. (Weimer, 1996). To promote host growth, these bacteria release energy stored in complex plant carbohydrates (Flint and Bayer, 2008), by converting them into short-chain fatty acids, vitamins, and other compounds. Meanwhile, the bacterial-released protein is also an important protein source for ruminants (Flint, 1997; Mizrahi et al., 2013). In addition, microorganisms secrete a large number of antagonistic factors (e.g., hydrogen peroxide, bacteriocins, diacetyl, etc.) that have a significant inhibitory effect on a wide range of bacteria (Cox and Dalloul, 2015). These antagonistic factors protect the organisms from various pathogenic bacteria and also reduce the colonization of potentially pathogenic bacteria. Subsequently, they help to maintain host health and normal physiological functions throughout the life cycle. Therefore, the development of beneficial rumen-derived microorganisms is of great significance to promote the development of the ruminant industry.

The ruminant gut microbiota is rich in microorganisms and the interactions between these microorganisms are complex and crucial to the host’s health. A growing body of research has highlighted that the gut microbiota and its metabolic activities with the host are essential in understanding nutrition and metabolism (Del Chierico et al., 2018; Valdes et al., 2018), of which the role of probiotics has, indeed, been emphasized. Additionally, it has been discovered that gut microbiota’s metabolic reactions help the body in nutrient absorption from the diet and transform them into a range of secondary metabolites to maintain gut health. The beneficial microorganisms ultimately cause widespread changes in intestinal metabolites, which in turn maintain the equilibrium in the intestinal metabolic microenvironment by carrying out a variety of metabolic functions in the gut. Additionally, these metabolites could also be utilized by the microorganisms for their proliferation (Foroutan et al., 2020). Host physiological activity supports intestinal homeostasis by lowering the amount of tryptophan and promoting indole derivatives that activate aryl hydrocarbon receptors (Williams et al., 2014). Conversely, toxins produced by intestinal microbes could potentially impact intestinal epithelial cells and result in intestinal injury (Kim et al., 2014).

Numerous studies found that microorganisms have been increasingly used as feed additives in ruminants. They achieve this by stimulating a shift in the harmful gut microbiota toward a healthier microbiota, improving feed utilization and daily weight gain (Marsalková et al., 2004; Sun et al., 2010). They promote *in vitro* fermentation and fiber degradation microbiota (Izuddin et al., 2018), affect body metabolism (Liu et al., 2017; Kim et al., 2018), and help to build better immune status. Moreover, they improve intestinal health (Izuddin et al., 2019) and even prevent diseases (Larsen et al., 2014). Hence, we hypothesized that ruminant probiotics achieve weight gain *via* strengthening intestinal microbiota/metabolites and based on intestinal microecology.

Until now, many swine and poultry studies have demonstrated that probiotics help to develop healthy microflora (mainly probiotics), which prevent pathogen adhesion and invasion of intestinal epithelial cells, induce the production of antibacterial compounds, maintain epithelial barrier integrity, and regulate metabolism and immune system (Wang et al., 2019; Šikić Pogačar et al., 2020; Chance et al., 2021). However, there is little evidence that ruminant-derived probiotics regulate gut microbial composition and thus affect metabolism to promote beef cattle body weight. Furthermore, most previous studies used a combination of strains (some not ruminant-derived) and primarily focused on the synergy of strains rather than the mechanism of a specific strain. This study aimed to isolate strains with fiber degradation potential and antibacterial ability from the buffalo rumen, evaluate their growth-promoting effects, investigate their influence on fecal flora and host metabolism, and analyze the impact of changes in intestinal flora on host metabolism. The gut microbiota, microbial metabolism, and potential probiotic effects were also investigated with possible mechanisms. In this study, the addition of a single probiotic seems a precise intervention, providing a meaningful reference for probiotic development in ruminants.

## Materials and methods

### Isolation and screening of cellulolytic bacteria

The modified cellulase identification medium (CMC-Na medium) was used as an isolation medium (Supplementary Table 1). The rumen fluid was obtained from the rumen of healthy buffaloes with rumen fistulas and filtered by four layers of sterilized gauze. The rumen fluid was diluted with sterilized double-distilled water, evenly coated on a CMC-Na medium, and then cultured in an anaerobic container at 39°C for 3 days. After that, the single colonies were selected and cultured on the CMC-Na medium. The culture mediums were dyed with 0.1% Congo red (Solarbio, China) staining solution to observe whether there were light yellow hydrolysis circles around the coating (Teather and Wood, 1982). The strains producing hydrolytic circles were selected for purification and subculture.

### Identification of cellulolytic bacteria

The isolated strains were confirmed and identified by genetic analysis using PCR and 16S rRNA sequencing for further verification. The genomic DNA was extracted with the bacterial genome DNA fast extraction kit (Aidlab Biotech Co., Ltd., China) according to the manufacturer’s protocol. Universal PCR primers 27F (5′-AGAGTTTTGATCCTGGCTCAG-3′) and 1492R (5′-GGTTACCTTGTTACGCACTT-3′) were used to amplify the 16S rRNA gene. PCR products were sequenced by Sangon Biotech Co., Ltd. (Shanghai, China). The sequencing results were analyzed using Basic Local Alignment Search Tool (BLAST) on the NCBI website. The phylogenetic tree of bacteria was constructed by the neighbor-joining method using MEGA7.0 software. The phylogenetic tree was statistically evaluated using 1,000 bootstrap replicates.

### Enzyme assay

Potato dextrose agar (PDA) medium-guaiacol (0.04% guaiacol), PDA-aniline blue medium (0.1 g/L aniline blue), Luria–Bertani (LB) plate (1% soluble starch), and an LB plate (1% skimmed milk) were, respectively, used to detect the laccase (Lac), manganese peroxidase (Mnp), lignin peroxidase (Lip), amylase, and protease in the strains.

### *In vitro* antibacterial test

The antibacterial activity of the isolates was determined by the Oxford cup method (Bian et al., 2016). *Escherichia coli* O157, O139, K88, K99, *Salmonella* C78-1, and *Staphylococcus aureus* ATCC25923 were used as an indicator at 1.0 × 10^7^ CFU/ml. These indicator bacteria were obtained from the State Key Laboratory of Agriculture Microbiology of Huazhong Agricultural University. In a super clean bench, the bacterial solution of the indicator bacteria (*Escherichia coli* O157, O139, K88, K99, *Salmonella* C78-1, and *Staphylococcus aureus* ATCC25923) was evenly coated onto solid LB plates, respectively. Sterilized Oxford cups (small round tubes with an inner diameter of 6 nm, an outer diameter of 8 nm, and a height of 10 nm) were then placed in the LB plates so that they were in contact with the LB plates without gaps. A 200 μL of SN-6 bacterial solution was added to the Oxford cup. The size of the inhibition zone was measured with a vernier caliper after overnight incubation.

### Tolerance test of heat, gastric juice, and intestinal fluid

The bacterial liquid (2.4 × 10^9^ CFU/ml) in the logarithmic growth phase was placed in a water bath at 70 and 90°C, respectively. Samples were taken at 3 and 10 min time points to count the viable bacteria in the samples.

Artificial gastric juice and intestinal juice (Yuanye Biotechnology Co., Ltd., Shanghai, China) were prepared according to the Chinese Pharmacopeia (D’Aldebert et al., 2009). The bacterial liquid (2.4 × 10^9^ CFU/ml) in the logarithmic growth phase was inoculated into artificial gastric juice (pH = 3.0) and artificial intestinal fluid (pH = 7.0) with 1% inoculation amount. Samples were taken at 3 and 4 h, respectively. Finally, the viable bacteria in the samples were counted.

The survival rate was calculated as follows: survival rate = [C/C_0_] × 100%. Here, C and C_0_ represented the number of colonies in the experimental and control groups, respectively.

### Antibiotic susceptibility assay

The drug sensitivity of isolated strains was tested with the disk diffusion method (Ghosh et al., 2015). Fifteen drug tablets (Hangzhou microbial Reagent Co., Ltd., China) were selected. The drug sensitivity detection was performed according to the latest version of the CLSI standard (Institute and Laboratory) (CLSI, 2018).

### Polymerase chain reaction amplification of virulence genes

*Bacillus cereus*, which contains nheA, nheB, nheC, and entFM genes was used as the positive control strain. The specific synthesized primers of virulence genes were obtained from Sangon Biotech Co., Ltd. (Shanghai, China). The amplification program was as follows: pre-denaturation at 94°C for 3 min; 35 cycles (95°C 3 min, 58°C 30 s, 72°C 33 s); and extended for 10 min at 72°C (Rowan et al., 2003).

### Animal toxicity test

All animal experiments were approved and reviewed by the animal welfare and research department, ethics committee, Huazhong Agricultural University, Wuhan, China (Approval number: HZAUMO-2019-047).

Twenty-three-week-old KM (Kun Ming) mice (an equal number of male and female subjects) were randomly divided into the experimental group and control group (*n* = 10). The mice in the experimental group were given SN-6 by gavage at 2.0 × 10^8^ CFU/day for 2 weeks, while mice in the control group were given the same volume of saline. Behaviors, hair loss, mental state, and general health of the reared mice were observed throughout 2 weeks. After 2 weeks, the mice were sacrificed using chloral hydrate as anesthesia, and the heart, spleen, liver, lung, and kidney were collected to detect organ index. *T*-test was used to analyze the data. *p* < 0.05 was considered statistically significant.

### Simmental growth-promoting test

Five-six-month-old healthy Simmental beef cattle (female) with the same genetic background and similar initial weight from Hubei Liangyou Jinniu animal husbandry technology Co., Ltd. (China, Hubei) were selected. The initial weights of Simmental are shown in Supplementary Table 2. Cattle (*n* = 66) were randomly divided into control group (*n* = 33) and experimental group (*n* = 33). There was no significant difference in the initial weight of Simmental between the two groups (*p* > 0.05). The feeding lasted for 33 days. To assess the long-term sustained effects of SN-6, we fed Simmental beef cattle with SN-6 for an additional 28 days. Both the control cattle and experimental cattle were fed with the basic diet (Supplementary Table 3) *ad libitum* during the experiment. The control cattle were given normal water, whereas, the experimental cattle were given water that contained SN-6 at 1.0 × 10^10^ CFU/day/individual. Before and at the end of the experiment, the cows were weighed at fasting. Data are expressed with mean ± SD, analyzed by one-way analysis of variance using SPSS 21.0 software, and *p* < 0.05 was considered statistically significant. Fresh fecal samples were collected from the rectum with sterile gloves at the end of the experiment and immediately stored in sterile centrifuge tubes. All samples were immediately frozen on dry ice and stored at –80°C for further analysis.

### Fecal microbiota analysis

Total DNA was extracted from fecal samples using an E.Z.N.A.^®^ soil Kit (Omega Bio-Tek, Norcross, GA, United States). The extracted DNA was qualitatively and quantitatively detected by 1% agarose gel electrophoresis and NanoDrop 2000 UV-vis spectrophotometer (Thermo Scientific, Wilmington, NC, USA). The V3–V4 region of 16S rRNA was amplified by PCR with specific primers 338F (5′-ACTCCTACGGGAGGCAGCAG-3′) and 806R (5′-GGACHTACHVGGGTWTCTAAT-3′) (PCR instrument: GeneAmp 9700, ABI, USA). The PCR products were recovered by 2% agarose gel and purified by AxyPrep DNA Gel Extraction Kit (Axygen Biosciences, Union City, CA, USA). QuantiFluor™-ST (Promega, USA) was used for quantitative analysis. The fecal microbial DNA fragments were sequenced by the Illumina Miseq platform (Illumina, San Diego, CA, USA). The quality control and splicing of the original data were carried out by using Trimmomatic and Flash software. After quality control, the sequences and fuzzy bases less than 50 bp were removed. UPARSE software (version 7.1^1^) was used to cluster the optimized sequences according to 97% similarity; UCHIME software was used to remove chimeras. The taxonomy of each 16S rRNA gene sequence was analyzed by the RDP Classifier algorithm^2^ against the Silva (SSU123) 16S rRNA database using a confidence threshold of 70%. Chao1, ACE, Shannon, and Simpson indices were used to reflect α diversity. The core fecal microbiota of each group was shown by the Venn diagram. In β diversity analysis, principal coordinate analysis (PCoA) was used to determine the difference in species composition among samples. According to the composition and sequence distribution of samples at each taxonomic level, the differences in species abundance between groups were compared and tested by the Student *t*-test. The *p* < 0.05 was considered to be statistically significant. Microbial biomarkers associated with particular interventions were identified through linear discriminant analysis (LDA) effect size (LEfSe), with an effect size threshold of 3.

### Fecal metabolomics analysis

The effects of SN-6 on the fecal metabolism in Simmental were assayed by LC-MS-based untargeted metabolomics. Fecal samples (50 mg) were accurately weighed, and the metabolites were extracted using a 400 μL of methanol: water (4:1, v/v) solution. The mixture was allowed to settle at –20°C and treated with high throughput tissue crusher Wonbio-96c (Shanghai Wanbo Biotechnology Co., Ltd.) at 50 Hz for 6 min, followed by vortexing for 30 s and ultrasound treatment at 40 kHz for 30 min at 5°C. The samples were placed at –20°C for 30 min to precipitate proteins. After centrifugation at 13,000 g at 4°C for 15 min, the supernatants were transferred to sample vials for LC-MS/MS analysis.

Ultra high performance liquid chromatography-mass spectrum (UHPLC-MS) analyses were performed using a Vanquish UHPLC system (Thermo Fisher, Germany) coupled with an Orbitrap Q Exactive™HF-X mass spectrometer (Thermo Fisher, Germany). Samples were injected onto a Hypesil Gold C18 column (100 mm × 2.1 mm, 1.9 μm; Thermo Fisher, Germany) using a 17-min linear gradient at a flow rate of 0.2 ml/min, and the column temperature was maintained at 40°C. The eluents for the positive polarity mode were eluent A (0.1% formic acid in water) and eluent B (Methanol). The eluents for the negative polarity mode were eluent A (5 mM ammonium acetate, pH 9.0) and eluent B (Methanol). The solvent gradient was set as follows: 2% B, 1.5 min; 2–100% B, 12.0 min; 100% B, 14.0 min; 100–2% B, 14.1 min; 2% B, 17 min. Q Exactive™HF-X mass spectrometer via electrospray ionization (ESI) interface was operated in positive/negative polarity mode with a spray voltage of 3.2 kV and capillary temperature of 320°C, sheath gas flow rate of 40 arb, and aux gas flow rate of 10 arb.

### Statistical analysis

All results were presented as mean ± standard deviation (SD). A multivariate statistical analysis was performed using ropls (Version 1.6.2^3^) R package from Bioconductor on Majorbio Cloud Platform.^4^ To obtain an overview of the metabolic data, an unsupervised method of principle component analysis (PCA) was used, and general clustering, trends, or outliers were visualized. Orthogonal partial least squares discriminate analysis (OPLS-DA) was used for statistical analysis to determine global metabolic changes between comparable groups. Variable importance in the projection (VIP) was calculated in the OPLS-DA model. The p-values were estimated with paired Student’s *t*-test on single-dimensional statistical analysis. The correlations between the key fecal microbiota and fecal metabolites were assessed by the Spearman’s correlation coefficient and were visualized on a heat map generated by the Python software (Version1.0.0). To clarify the changes in metabolic pathways caused by SN-6 interventions, we characterized potential pathway enrichment analysis using the KEGG pathway.^5^

## Results

### Cellulolytic bacteria were isolated and identified

**FIGURE 1:**
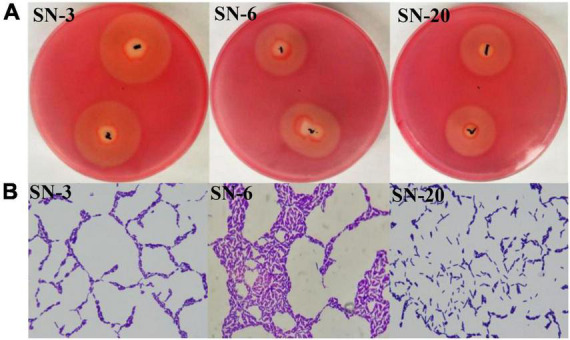
Isolation and identification of cellulolytic bacteria. **(A)** Diameter of hydrolysis circle. 1 and 2 represent the same strain. **(B)** Gram staining of strains (1,000×).

Based on the light yellow hydrolysis circle given on the CMC-Na agar plate, three strains named SN-3, SN-6, and SN-20 were isolated (Figure 1A), and all of them were gram-positive bacteria (Figure 1B). According to the enzyme activity (EA) value (Table 1), the degradation capacity of fiber was SN-3 = SN-20 > SN-6. The 16S rDNA sequence analysis indicated that SN-3 had 99.79% homology to *Bacillus pumilus*, SN-6 had 99.65% homology to *Bacillus paralicheniformis*, and SN-20 had 99.79% homology to *Bacillus altitudinis*. These results demonstrated that SN-3, SN-6, and SN-20 were *Bacillus pumilus*, *Bacillus paralicheniformis*, and *Bacillus altitudinis*, respectively.

**TABLE 1:** Enzyme activity (EA) index of SN-3, SN-6, and SN-20.

Strain	Diameter of hydrolysis circle (D, mm)	Diameter of lawn (d, mm)	Enzyme activity index (D/d)
SN-3	33.25	10.75	3.10
SN-6	24.80	10.16	2.44
SN-20	24.56	7.87	3.12

### Characteristics of enzyme production and antibacterial activity *in vitro*

All three strains could produce amylase, among which SN-6 is the best amylase producer. Both SN-3 and SN-20 could produce protease, and SN-3 was comparatively better than SN-20 (Table 2). Only SN-6 exhibited a reddish-brown oxidation circle on the PDA-guaiacol plate (Supplementary Figure 1), which indicated that SN-6 secreted laccase. None of the three strains could discolor PDA-aniline blue, suggesting that none of them produced Mnp and Lip (data not shown). SN-6 robustly inhibited K99 and *S. aureus* growth. The inhibition zone diameter was 22.0 mm for *E. coli* K99 and 24.0 mm for *S. aureus*. Moreover, SN-3 and SN-20 did not obviously inhibit six indicator bacteria (Supplementary Figure 2).

**TABLE 2:** Amylase/protease activity of strains.

Strain	Diameter of fading circle (mm)	Diameter of clear zone (mm)
SN-3	10.0	21.7
SN-6	24.0	0.0
SN-20	10.0	15.0

### Tolerance properties of SN-6 at different conditions

A probiotic with a higher tolerance capability and a good survival rate is often preferred. We found that the survival rate of SN-6 was 91.7% at 70°C for 3 min, and 58.3% at 90°C for 10 min. After culturing SN-6 in simulated gastric juice (pH 3.0) or in neutral simulated intestinal fluid for 4 h, its survival rate was 36.36 and 54.29%, respectively. These results indicated the good survivability of SN-6 in harsh environments.

### Safety assessment of SN-6

**FIGURE 2:**
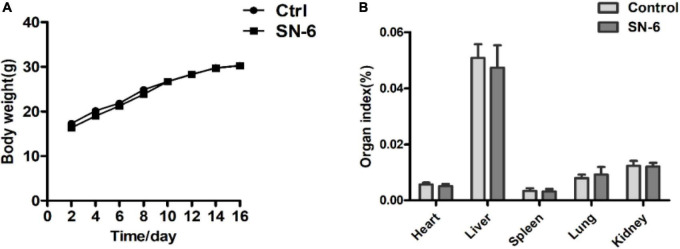
Body weight and organ index of mice in the control and SN-6 treated groups. The values are presented as the mean ± SD (*n* = 10). **(A)** Body weight and **(B)** organ index.

SN-6 was sensitive to antibiotics used in this study except for oxacillin and ceftazidime (Table 3). Enterotoxin-related virulence genes nheA, nheB, nheC, and entFM were amplified in the positive strain (Supplementary Figure 3), while no enterotoxin-related virulence gene was detected in SN-6 (Supplementary Figure 4). It was also observed that the mice, both in the control group and the experimental group, were normal. There was no significant difference in body weight (Figure 2A) and organ index between the two groups (Figure 2B).

**TABLE 3:** Antibiotic susceptibility test for SN-6.

Antibiotics	Bacteriostatic zone (mm)	Bacteriostatic effect
Penicillin	22.51	S
Ampicillin	13.24	M
Piperacillin	34.17	S
Oxacillin	0	R
Cephalosporin	37.84	S
Ceftazidime	0	R
Ceftriaxone	14.89	M
Ciprofloxacin.	26.16	S
Ofloxacin.	31.87	S
Norfloxacin.	22.66	S
Kanamycin.	25.34	S
Gentamicin.	24.81	S
Tetracycline.	25.76	S
Compound sulfamet-hoxazole (SMZ)	37.77	S
Clindamycin	18.94	S

### Growth promoting performance

**FIGURE 3:**
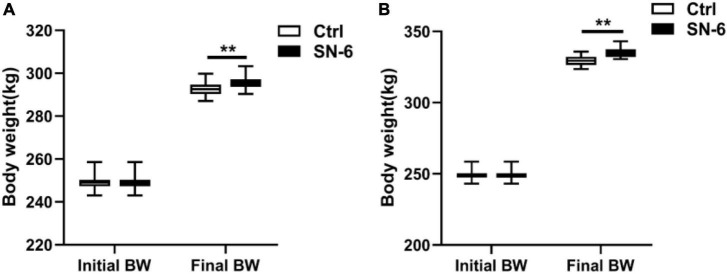
Body weight of Simmental in the control and SN-6 treated groups. **(A)** Feeding for 33 days. **(B)** Feeding for 61 days. The values are presented as the mean ± SD (*n* = 33). **p < 0.01 as statistically significant.

After 33 days of feeding, Simmental cattle’s body weight in the control and experimental groups was 292.53 and 295.86 kg, respectively. After 61 days of feeding, the body weight of Simmental cattle in the control and experimental groups was 329.48 and 335.62 kg, respectively. It was noted that the SN-6 feeding increased body weight by approximately 3.33 kg/individual (33 days), and 6.14 kg/individual (61 days) compared with the control (*p* < 0.01) (Figure 3).

### The regulation of SN-6 on fecal microbiota

A total of 475,965 optimized sequences of 16S rRNA of bacteria in 12 fecal samples (six in each group) were obtained. According to 97% sequence similarity, the optimized sequences were clustered by operational taxonomic units (OTU) and 1,338 OTU sequences were obtained. There was no significant difference in the α diversity index between the SN-6 group and the control group (*p* > 0.05), indicating that SN-6 feeding did not change the fecal flora richness and diversity (Supplementary Figure 5).

**FIGURE 4:**
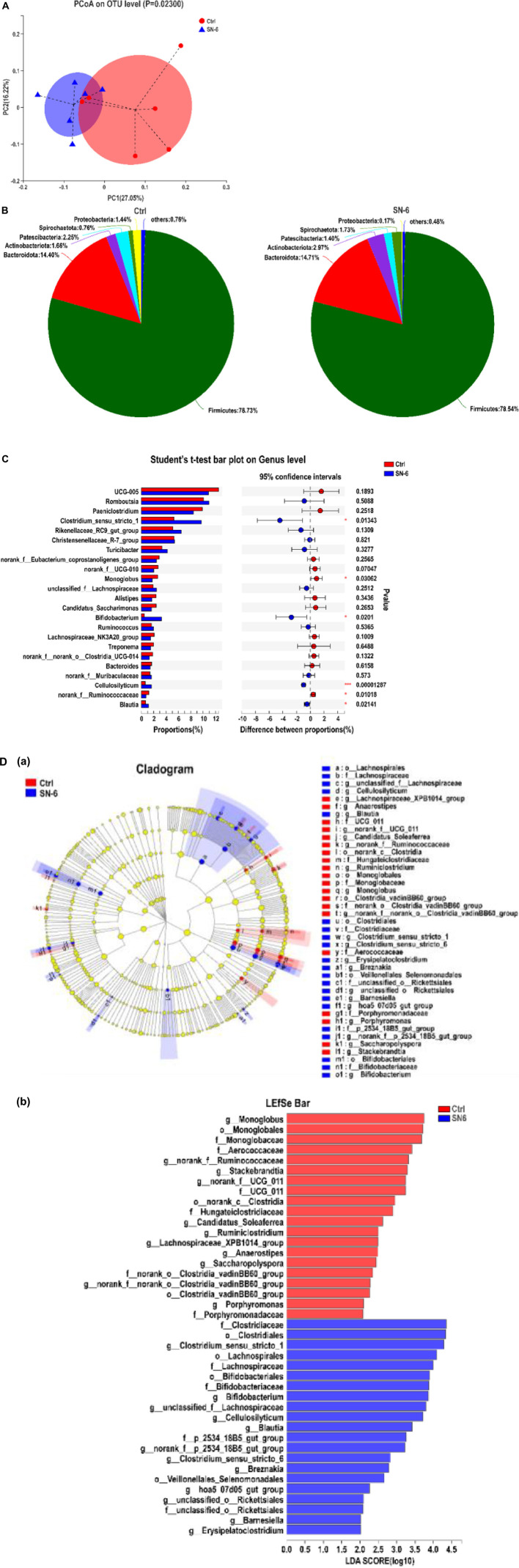
SN-6 alters the fecal microbiota composition of Simmental. **(A)** PCoA score plot of fecal microbiota based on OTU abundance. **(B)** Bacterial taxonomic profiling of fecal microbial community at phylum level. **(C)** Significant analysis of species difference at the genus level. *p < 0.05, **p < 0.01, ***p < 0.001. **(D)** Diversity analysis of LEfSe multistage species. (a) Cladogram. The circles radiating from the inside to the outside represent the taxonomic level from phylum to genus. Each small circle represents a classification at the same level, and the diameter of the small circle is positively related to its abundance. The species without significant differences are uniformly colored yellow. The red node represents the relatively important microbe in the control group, and the blue node represents the relatively important microbe in the SN-6 group. The species name is present on the right. (b) Histogram of LDA value distribution. The species with LDA score greater than 3.0 are biomarkers with a statistical difference. The length of the histogram represents the influence of the species with a significant difference.

Likewise, the beta diversity was assessed by principal coordinate analysis (PCoA) based on the Bray–Curtis distance, which was used to study the similarity or differences in sample community composition. As shown in Figure 4A, SN-6 significantly changed the overall community composition of fecal flora compared with the control (*p* < 0.05), which indicated that the microorganisms in the SN-6 group had distinct clustering.

At the phylum level, the fecal microbiota composition of each group is shown in Figure 4B. Firmicutes and Bacteroidetes are the core bacteria with high abundance in ruminants both in the SN-6 group and control group. However, SN-6 feeding increases the abundance of Actinobacteria, which might be due to the increase of *Bifidobacterium*. At the genus level, significantly increased abundances of *Clostridium_sensu_stricto_1*, *Bifidobacterium*, *Blautia*, and *Cellulolyticum* were observed in the SN-6 group (*p < 0.05, ***p < 0.001, respectively), while significantly decreased abundances of *Monoglobus*, norank_f*_Ruminococcacea* (*p < 0.05) (Figure 4C) were observed. Using LDA and LEfSe analyses for microbial biomarker discovery in two groups, *Clostridiaceae*, *Bifidobacteriaceae*, and *Lachnospiracese* were found enriched in the SN-6 group, while *Stackebrandtia* and *Monoglobus* were enriched in the control group (Figure 4D).

### The effects of SN-6 on fecal metabolism

**FIGURE 5:**
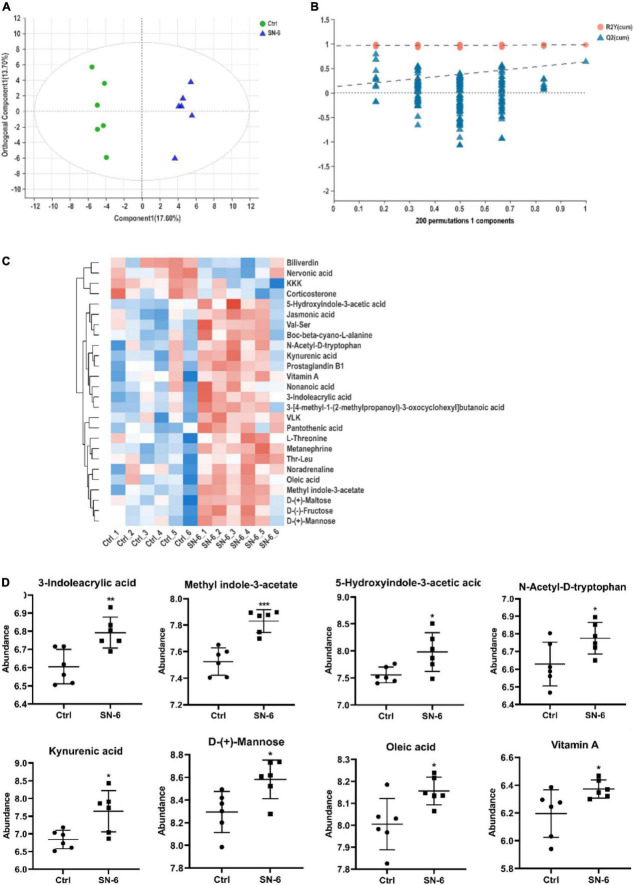
Effects of SN-6 on the fecal metabolism of Simmental. **(A)** The orthogonal projection to latent structures discriminant analysis (OPLS-DA) score plot between the control and SN-6 groups. **(B)** Two hundred times permutations of OPLS-DA plot between the control and SN-6 groups. **(C)** Heatmap overview of 26 differential metabolites in feces throughout control and SN-6 groups. **(D)** Effect of SN-6 on the abundance of potential metabolites in fecal samples. Data are shown as mean ± SD (*n* = 6). *p < 0.05, **p < 0.01, ***p < 0.001.

To get a holistic view of the host metabolism after SN-6 intervention, we used non-targeted metabolomics to identify key metabolites and metabolic pathways that might be altered in the Simmental intestine. A total of 185 metabolites were identified in feces. The OPLS-DA score scatter plots revealed a visible separation between the control and SN-6 groups in positive ion mode (R^2^Y: 0.982, Q^2^: 0.635) (Figure 5A). The results of 200 permutations exhibited no over-fitting in OPLS-DA models (Figure 5B). Twenty-six metabolites were found in the fecal sample which met the conditions of *p* < 0.05 and variable importance in project (VIP) > 1 between the control and SN-6 groups (Figure 5C). The effect of SN-6 on the regulation of some differential metabolites (including 3-indoleacrylic acid, 5-hydroxyindole-3-acetic acid, methyl indole-3-acetate, *N*-acetyl-*D*-tryptophan, oleic acid, *D*-mannose, vitamin A, and kynurenic acid) in the feces is shown in Figure 5D.

### Putative metabolic pathways related to SN-6 interventions

**FIGURE 6:**
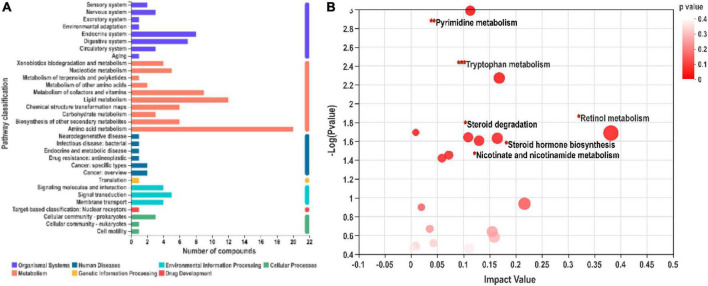
Kyoto encyclopedia of genes and genomes (KEGG) functional pathway annotation **(A)** and enrichment **(B)** analysis. **(A)** The ordinate represents the secondary classification items of the KEGG pathway, and the abscissa represents the number of metabolites annotated to the pathway. **(B)** Pathway enrichment analysis of differentially expressed metabolites.

As shown in Figure 6A, differential metabolites related to different metabolic pathways were mapped. Six potential metabolic pathways were screened according to impact value > 0.1 and *p* < 0.05. Retinol metabolism (0.38), tryptophan metabolism (0.17), steroid hormone biosynthesis (0.16), nicotinate and nicotinamide metabolism (0.13), pyrimidine metabolism (0.11), and steroid degradation (0.11) are listed in descending order of impact value (Figure 6B). Among them, tryptophan metabolism covered the main differential metabolites, indicating that this pathway might play a vital role in the growth promotion of SN-6.

### Associations between key fecal microbiota and fecal metabolites

**FIGURE 7:**
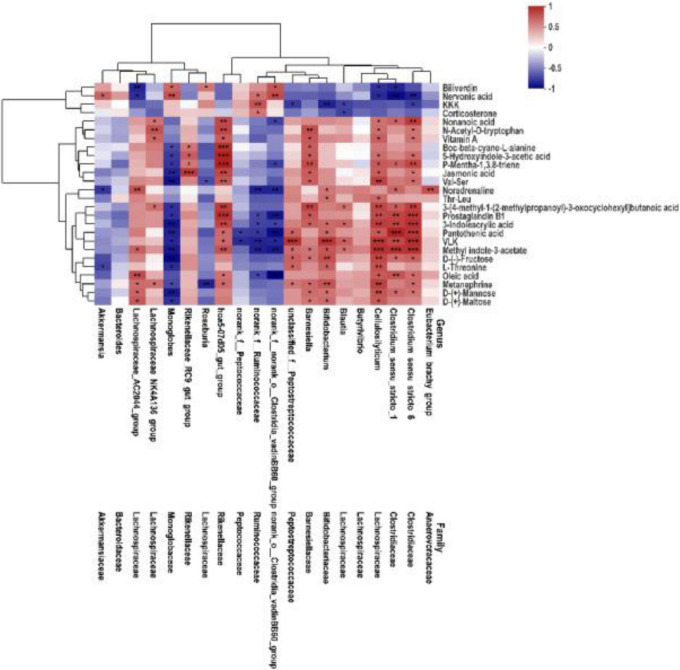
Association heatmap of Spearman’s correlations analyses integrating key microbes and metabolome of fecal. The color scale represents the Spearman’s R-value (red, positive correlation; blue, negative correlation). *p < 0.05, **p < 0.01, and ***p < 0.001.

To comprehensively analyze the relations between fecal metabolites and gut microbiota, weight gain associated with altered metabolites was explored. Spearman’s correlation analysis was performed to determine the association between key fecal microbiota and differential metabolites. As shown in Figure 7, indole derivatives (including 3-indoleacrylic acid, methyl indole-3-acetate, 5-hydroxyindole-3-acetic acid), lipids (including vitamin A, oleic acid), and amino acids/peptides (including Val–Ser, L-threonine) were positively correlated with f_*Clostridiaceae*, f_*Lachnospirceae* (except for Roseburia), g_*Bifidobacterium*, unclassified_f*_Peptostreptococcaceae*, g_*Barnesiella*, and f_*Rike nellaceae*, and were negatively correlated with norank_ f_norank_o_*Clostridia_vadinBB60_group*, norank_f_*Ruminoco ccaceae*, and *Monoglobus*. These relations suggested that fecal microbiota could affect fecal metabolites in SN-6-fed Simmental.

## Discussion

The rumen is the unique digestive organ of ruminants, and there are about 10^10^ bacteria per gram of rumen contents. Among them, cellulolytic bacteria are considered to be the main cellulose-decomposing agents (Brulc et al., 2009), accounting for about 10% of rumen microorganisms (Russell et al., 2009). The digestion and absorption of crude fiber in ruminants feed are completely dependent on rumen microorganisms, which exhibit a complex interplay with the host’s functions. Rumen bacteria significantly contribute to food digestion, thus considered potent probiotics and transferring these bacteria to other beef cattle could increase their daily weight gain. That is why, this study aimed to isolate and identify such kinds of bacteria and transfer them to the other cattle to investigate the daily weight gain and their interrelationship with host gut microecology and metabolic homeostasis. Three bacterial strains (*Bacillus pumilus* SN-3, *Bacillus paralicheniformis* SN-6, and *Bacillus altitudinis* SN-20, respectively) were isolated from the rumen of buffaloes. By evaluating SN-3, SN-6, and SN-20 for their antimicrobial properties, the ability to produce fiber digesting enzymes and proliferation, we selected the outstanding SN-6 and focused its possible effects on the Simmental cattle. Typically, SN-6 secrets laccase, which could degrade lignin in crude fiber and releases cellulose from the lignin. Most probably, it significantly contributes to the growth performance of the Simmental.

Previous studies have confirmed that rumen microorganisms could secrete antibacterial substances, which could effectively inhibit the growth and multiplication of various pathogenic bacteria (Ren et al., 2019). It is also described that SN-6 had strong inhibitory effects on pathogenic *S. aureus* and *E.coli* K99. Therefore, it is reasonable to speculate that SN-6 is capable of preventing diseases caused by pathogenic *S. aureus* and *E. coli* K99, such as cow mastitis (Azara et al., 2017), calf diarrhea (Yadegari et al., 2019), piglet diarrhea (Xia et al., 2018), and so on. Interestingly, SN-6 strongly inhibited both gram-negative and gram-positive pathogenic bacteria, suggesting that SN-6 was a promising alternative for food antibiotics in livestock. In addition, the number of living bacteria of SN-6 after fermentation was up to 190 billion/g (data not shown), which demonstrated its tremendous advantage of convenience in preparation.

It was reported that probiotics contribute to nutrition and metabolic health (Koh et al., 2016). Cox et al. demonstrated that probiotics promote a stable intestinal microbiota, stimulate digestive rates, and improve intestinal nutritional health (Cox and Dalloul, 2015). Possibly, these probiotics produce a large number of active enzymes during nutrient metabolism, which in turn increase intestinal digestive enzyme activity and promote nutrient absorption (Hu et al., 2018; Gong et al., 2018; Cao et al., 2020). In addition, numerous studies have shown that probiotics improve the feeding efficiency of animals by regulating the intestinal flora, and promoting the growth of dairy cows, lambs, rabbits, and sows (Sun et al., 2013; Jia et al., 2018; Liu L. et al., 2019; Zhang et al., 2020). Various nutrients ingested by the organisms are metabolized by a wide range of gut microbes to maintain complex life activities, where metabolites are transported, absorbed, or excreted through highly dynamic metabolic pathways (Maurice et al., 2013; Lamichhane et al., 2018). Importantly, the produced intestinal metabolites (i.e., tryptophan and short-chain fatty acids) nourish intestinal epithelial cells (Flint, 2016), improve the intestinal lining [He et al., 2022 (Microbiome)], and regulate downstream signaling pathways (Gill et al., 2006; Flint, 2016), acting as a link between the gastrointestinal tract and host health. The current study found that SN-6 could significantly regulate the intestinal flora and increase the average daily weight gain of Simmental, which is consistent with the previous studies. However, the mechanism of promoting growth by probiotics is not completely understood. Therefore, the effects of SN-6 feeding on fecal microbiota and metabolism of Simmental were explored in the present study, and the possible association between fecal microbiota and metabolism was evaluated.

The intestinal microbiota has irreplaceable importance in the host’s vital activities and is, therefore, also known as “another organ of the body” (de Vos et al., 2022). The dynamics of the microbiota are influenced by diet, the environment, and other conditions. Probiotic intervention could alter the abundance and composition of microbiota in the gut, which in turn could affect host health (Schepper et al., 2019; Wang et al., 2020). The influences of gut microbiota on the host are highly correlated with complex interactions involving the host–microbe axes series (Xie et al., 2013). Studies on the gut microbiota provide a reference to explore the impact of gut microbiota interactions on organismal health. In this study, we also found that SN-6 feeding significantly influenced β diversity of the host gut microbiota, indicating that SN-6 had significant effects on microbial community structure in the host gut. Also, the differences in specific microorganisms further visualized the intrinsic link between SN-6 addition and gut microbiota composition. The results of this study revealed that SN-6 could increase the relative abundance of potentially beneficial bacteria (i.e., *Clostridiaceae*, *Lachnospirales*, and *Bifidobacteriales*) (Figure 4E), which most probably played an important role in promoting nutrient absorption, preventing diseases, and maintaining host health. *Clostridium* is a beneficial bacterium against intestinal bacterial infection (Behnsen, 2017; Kim et al., 2017). *Lachnospira* has a considerable ability to utilize dietary polysaccharides (O Sheridan et al., 2016). Similarly, members of *Bifidobacterium* are considered to play a critical role in maintaining human health (Di Gioia et al., 2014; Kusada et al., 2017). Meanwhile, the regulation of *Bifidobacterium* by SN-6 constituted the main factor underlying the increase in phylum *Actinobacteria*. The above results confirm that SN-6 feeding led to the development of a better structure of the host gut microbiota. In contrast, *Monoglobus* and *Stackbrandtia* were enriched in the control group. *Monoglobus* is often seen in an abnormal inflammatory state and tends to be elevated in the disease groups (Zhang et al., 2021; Miao and Davies, 2010). There are few studies and reports on the function of *Stackbrandtia*, which belongs to the *Actinobacteria*, *Glycomycetaceae*, and is mostly of environmental and soil origin (Zhang et al., 2016; Liu et al., 2018). Although all cattle were healthy, health-threatening microorganisms were still shown to be enriched in the gut of the control group, and these bacteria are likely to be transformed into pathogenic bacteria and involved in intestinal bacterial dysbiosis and disease transmission. Therefore, it is reasonable to speculate that SN-6 increased beneficial bacteria that promote nutrient absorption and helped in disease prevention and inhibited the colonization of potentially harmful bacteria, thus playing a significant role in increasing daily weight gain in Simmental by regulating the intestinal flora. Gut microbes perform a diverse range of metabolic functions including the production of numerous metabolites (Valdes et al., 2018). Increasingly recognized metabolites produced by gut microbes are vital mediators of diet-induced host–microbe interactions. We found that SN-6 affected the fecal metabolic pathways and metabolite concentrations of Simmental, amino acid metabolism, lipid metabolism, and vitamin metabolism were more enriched in the SN-6 group. Moreover, the contents of certain indole derivatives, lipids, and amino acids/peptides in the SN-6 group were significantly higher than those in the control group (Figure 6A). Amino acids are essential precursors for the synthesis of proteins and peptides and have been identified as markers of protein metabolism (Liu C. et al., 2019). Indole acrylic acid plays an essential role in maintaining intestinal homeostasis and barrier integrity (Agus et al., 2018). Kynurenic acid, produced by tryptophan metabolism, might have anti-inflammatory properties in the gastrointestinal tract and participate in immune regulation (Kennedy et al., 2017). In the metabolomic data, we also observed higher oleic acid and mannose contents in the SN-6 group. Oleic acid has natural antioxidant and anti-inflammatory properties. Zhang et al. (2017) found that mannose has an immunomodulatory function, which could specifically induce the differentiation of naive T cells into regulatory T cells (Treg). In mouse models, oral mannose could prevent and inhibit certain autoimmune diseases (Zhang et al., 2017). These findings indicate that SN-6 might exert a growth-promoting effect by elevating the relative concentrations of some positive functional metabolites that promote organismal health and homeostasis.

The composition and metabolic pattern of the host–gut microbiota gradually change with the intervention of probiotics (Nealon et al., 2017). In this study, there was a significant correlation between fecal microbes and metabolites (Figure 7). Indole derivatives (including 3-indoleacrylic acid, methyl indole-3-acetate, 5-hydroxyindole-3-acetic acid), lipids (including vitamin A, oleic acid), and amino acids/peptides (including Val–Ser, L-threonine) were positively correlated with f_ *Clostridiaceae*, f_ *Lachnospiraceae* (except for Roseburia), g_*Bifidobacterium*, unclassified_f_*Peptostreptococcaceae*, g_*Bar nesiella*, f_ *Rikenellaceae*, and were negatively correlated with norank_f_norank_o_*Clostridia_vadinBB60_group*, norank_f_ *Ruminococcaceae*, and *Monoglobus*. Many studies have reported that *Clostridiaceae*, *Bifidobacterium*, and *Peptostreptococcaceae* could convert tryptophan into indole and indole derivatives (Aragozzini et al., 1979; Wikoff et al., 2009; Russell et al., 2013; Williams et al., 2014; Dodd et al., 2017; Wlodarska et al., 2017). Studies have shown that tryptophan and its downstream metabolites could bind to aryl hydrocarbon receptor (AHR); the resulting complex is transported into the nucleus, where AHR is activated (Lanis et al., 2017) to regulate intestinal homeostasis, improve gut barrier function, and activate the immune system (Roager and Licht, 2018). These findings also confirmed that SN-6 feeding altered the composition and metabolic pattern of the intestinal flora and thus contributed to the daily weight gain of Simmental.

It should be mentioned that additional work is needed to address some limitations of the present study, e.g., verification experiments, and some of the work, such as whether SN-6 modulates gut and rumen microflora metabolism and influences the immunity of the body, is under investigation in our research group.

## Data availability statement

The datasets presented in this study can be found in online repositories. The names of the repository/repositories and accession number(s) can be found in the article/Supplementary material.

## Ethics statement

The animal study was reviewed and approved by the ethics committee of animal welfare and research department, Huazhong Agricultural University.

## Author contributions

DS, YX, SY, and JL: conceptualization. DS, SY, JL, YC, YX, and ZZ: methodology. SY: software, data curation, and writing – original draft preparation. SY and RW: formal analysis. SY, HL, MA and DS: writing – review and editing. DS: supervision. All authors had read and approved the final manuscript.

## Footnotes

1
http://drive5.com/uparse/

2
http://rdp.cme.msu.edu/

3
http://bioconductor.org/packages/release/bioc/html/ropls.html

4
https://cloud.majorbio.com

5
https://www.genome.jp/kegg/pathway.html
