# Supplementary material for: A buffalo rumen-derived probiotic (SN-6) could effectively increase simmental growth performance by regulating fecal microbiota and metabolism
Source: Front Microbiol. 2022 Oct 28;13:935884. doi: 10.3389/fmicb.2022.935884 (PMC9649902; doi:10.3389/fmicb.2022.935884)
Supplement: Supplementary file 1 [file Data_Sheet_1.doc]

Supplementary Material

# Supplementary Figures and Tables

## Supplementary Figures


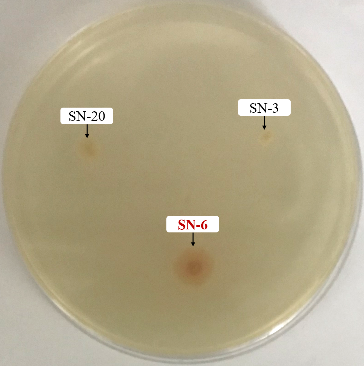


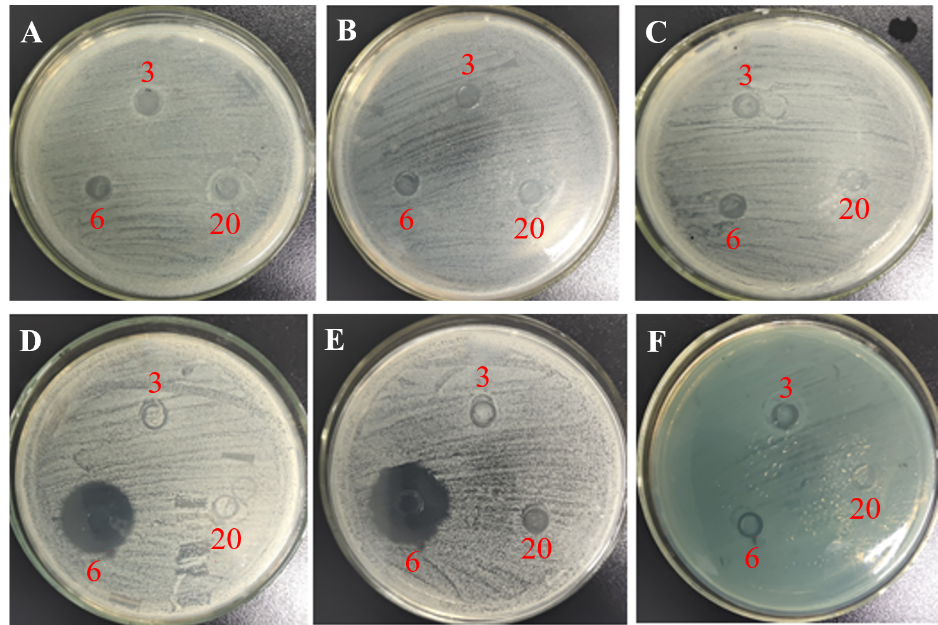


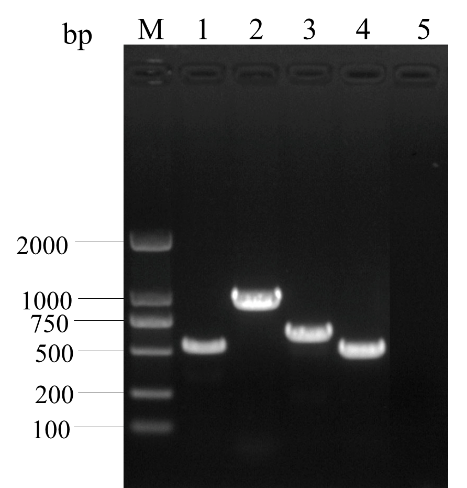


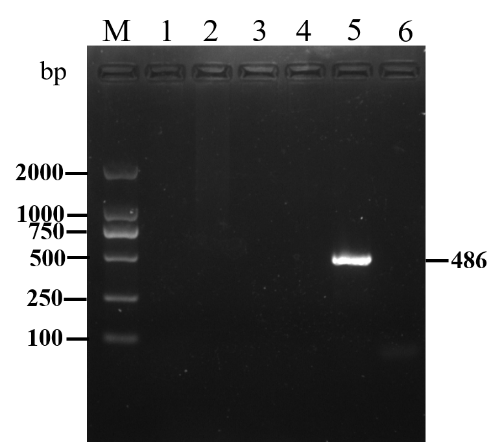


**A**

**B**


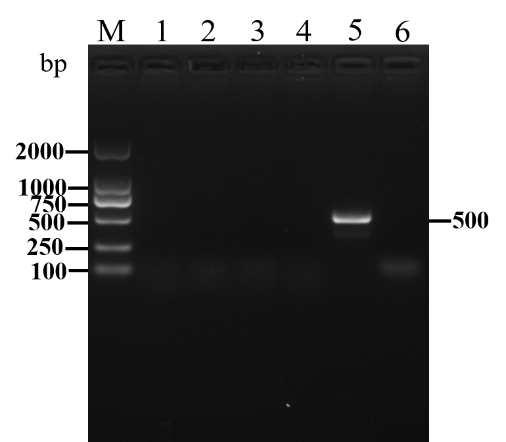


**D**


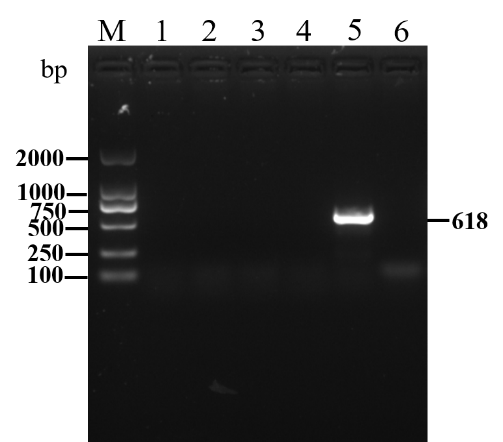

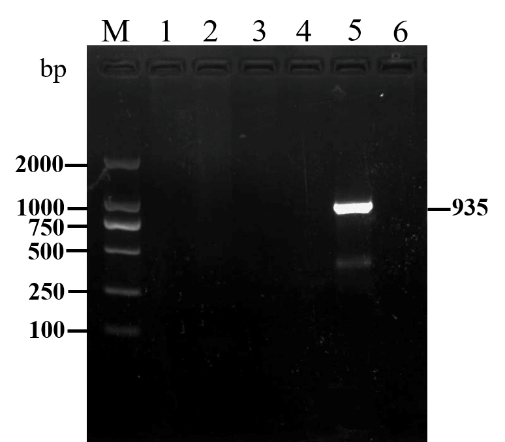


**C**


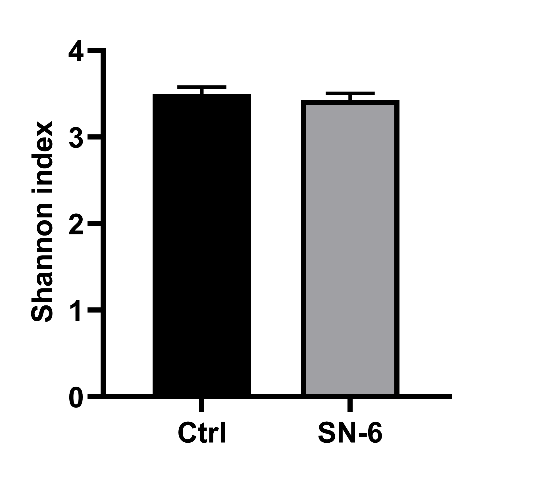


**A**


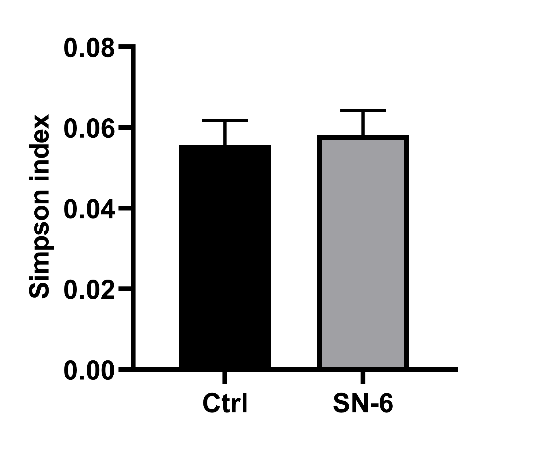


**B**


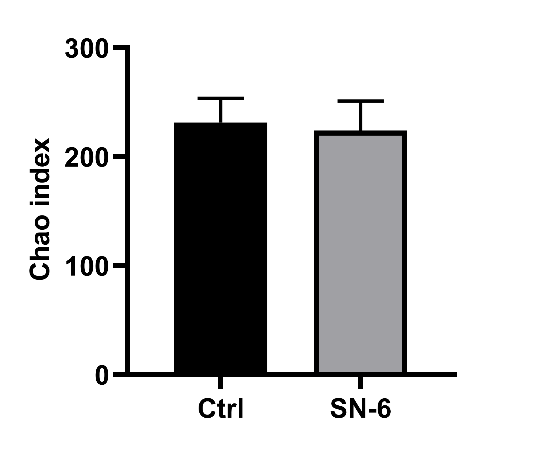


**C**


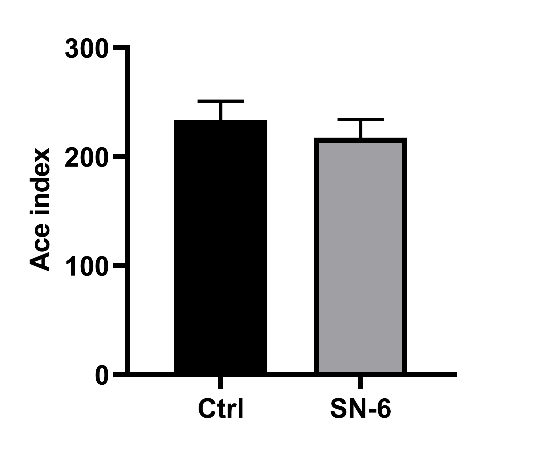


**D**

**Supplementary Figure 1.** Growth of strains on PDA-guaiacol plate.

**Supplementary Figure 2.** The results of antibacterial experiment. The inhibition zone diameter of strains against E. coli O157 (A) O139 (B) K88 (C) K99 (D) S. aureus (E) S. enteritidis (F). 3, 6, 20 represent SN-3, SN-6 and SN-20, respectively.

**Supplementary Figure 3.** The virulence gene of Bacillus cereus was amplified by PCR. M: DL2000 DNA molecular weight standard; Lane 1: nheA; Lane 2: nheB; Lane 3: nheC; Lane 4: entFM; Lane 5: negative control.

**Supplementary Figure 4.** Detection of SN-6 virulence genes. M: DL2000 DNA molecular weight standard; Lane 1: SN-20; Lane 2: SN-6; Lane 3: SN-4 strain preserved in our laboratory; Lane 4: SN-3; Lane 5: positive control strain; Lane 6: negative control. (A) entFM (B) nheA (C) nheB (D) nheC

**Supplementary Figure 5.** Comparison of alpha diversity as assessed by the (A) Shannon (B) Simpson (C) Chao (D) Ace indices.

## Supplementary Tables

**Supplementary Table S1.** The only carbon source plate formula of sodium carboxycellulose (L-1 distilled water).

| Name | Usage |
| --- | --- |
| CMC-Na | 4.0 g |
| peptone | 1.0 g |
| agar | 10.0 g |
| buffer A | 165 mL |
| buffer B | 165 mL |

Buffer A (L-1 distilled water): 3.0 g (NH4)_2_SO_4_, 3.0 g KH_2_PO_4_, 6.0 g NaCl, 0.4 g CaCl_2_·2H_2_O, 0.6 g MgSO_4_·7H_2_O. Buffer B (L-1 distilled water): 4.0 g K_2_HPO_4_·3H_2_O. The pH of all medium was adjusted to 7.0 before sterilization at 121 °C for 15 min.

**Supplementary Table S2-1.** The initial body weight of Simmental.

**Supplementary Table S2-2.** The final body weight of Simmental.

| Serial number | Initial weight (kg) | |
| --- | --- | --- |
|  | the control group | the SN-6 group |
| 1 | 244.0 | 247.5 |
| 2 | 248.5 | 248.5 |
| 3 | 243.0 | 247.0 |
| 4 | 253.5 | 249.0 |
| 5 | 258.5 | 248.5 |
| 6 | 247.0 | 249.5 |
| 7 | 248.0 | 251.0 |
| 8 | 246.5 | 246.5 |
| 9 | 250.0 | 250.0 |
| 10 | 249.5 | 248.0 |
| 11 | 251.0 | 249.5 |
| 12 | 249.5 | 253.5 |
| 13 | 249.0 | 249.5 |
| 14 | 247.5 | 250.0 |
| 15 | 249.0 | 247.5 |
| 16 | 248.5 | 248.5 |
| 17 | 250.0 | 243.0 |
| 18 | 248.5 | 254.5 |
| 19 | 249.5 | 251.0 |
| 20 | 247.5 | 249.0 |
| 21 | 248.5 | 245.5 |
| 22 | 249.5 | 244.0 |
| 23 | 252.0 | 245.0 |
| 24 | 251.0 | 52.0 |
| 25 | 254.5 | 246.0 |
| 26 | 245.5 | 259.5 |
| 27 | 250.5 | 249.5 |
| 28 | 246.0 | 258.5 |
| 29 | 245.0 | 248.5 |
| 30 | 247.5 | 246.0 |
| 31 | 251.5 | 252.5 |
| 32 | 248.5 | 247.0 |
| 33 | 249.5 | 251.0 |

| Serial number | Final weight of 33 days(kg) | | Final weight of 61 days(kg) | |
| --- | --- | --- | --- | --- |
|  | the control group | the SN-6 group | the control group | the SN-6 group |
| 1 | 287.8 | 290.3 | 323.6 | 334.0 |
| 2 | 287.0 | 293.1 | 324.0 | 331.5 |
| 3 | 289.8 | 291.8 | 325.5 | 335.5 |
| 4 | 290.7 | 293.9 | 332.0 | 331.0 |
| 5 | 290.4 | 295.1 | 334.5 | 332.0 |
| 6 | 290.6 | 296.4 | 327.3 | 335.5 |
| 7 | 290.4 | 295.1 | 333.5 | 335.9 |
| 8 | 290.2 | 294.3 | 328.5 | 338.8 |
| 9 | 289.5 | 293.1 | 325.5 | 337.5 |
| 10 | 294.3 | 296.1 | 335.8 | 337.5 |
| 11 | 289.4 | 293.0 | 336.0 | 335.0 |
| 12 | 294.3 | 293.7 | 327.0 | 343.0 |
| 13 | 289.4 | 296.2 | 334.0 | 337.0 |
| 14 | 293.7 | 293.7 | 326.5 | 330.5 |
| 15 | 292.6 | 293.1 | 329.9 | 331.9 |
| 16 | 292.3 | 294.6 | 332.0 | 337.0 |
| 17 | 296.1 | 296.1 | 329.5 | 331.0 |
| 18 | 291.5 | 295.8 | 331.0 | 340.0 |
| 19 | 294.3 | 296.8 | 332.5 | 342.0 |
| 20 | 291.1 | 296.3 | 325.0 | 333.2 |
| 21 | 290.9 | 297.3 | 329.5 | 335.0 |
| 22 | 293.0 | 293.8 | 326.5 | 334.5 |
| 23 | 292.8 | 297.5 | 334.2 | 331.5 |
| 24 | 296.9 | 296.9 | 331.2 | 339.0 |
| 25 | 294.4 | 300.5 | 329.5 | 336.5 |
| 26 | 296.5 | 299.3 | 329.0 | 337.2 |
| 27 | 295.8 | 301.9 | 328.5 | 336.0 |
| 28 | 295.0 | 300.8 | 326.0 | 341.5 |
| 29 | 299.8 | 303.3 | 328.0 | 332.0 |
| 30 | 291.4 | 291.5 | 328.5 | 330.0 |
| 31 | 295.8 | 297.9 | 333.5 | 336.5 |
| 32 | 291.8 | 296.0 | 328.5 | 337.5 |
| 33 | 291.0 | 298.2 | 326.2 | 338.0 |

Supplementary Table S3. Basic diet formula of Simmental.

| Feed ingredient | Proportion (%) |
| --- | --- |
| corn silage | 60.87 |
| wheat straw | 6.61 |
| spent mushroom substrate | 19.72 |
| concentrated feed | 12.79 |
